# Editor's cut: DNA cleavage by CRISPR RNA-guided nucleases Cas9 and Cas12a

**DOI:** 10.1042/BST20190563

**Published:** 2019-12-24

**Authors:** Thomas Swartjes, Raymond H.J. Staals, John van der Oost

**Affiliations:** Laboratory of Microbiology, Wageningen University & Research, Stippeneng 4, 6708 WE Wageningen, Netherlands

**Keywords:** biotechnology, CRISPR, DNA binding, endonucleases, genome editing, non-coding RNA

## Abstract

Discovered as an adaptive immune system of prokaryotes, CRISPR–Cas provides many promising applications. DNA-cleaving Cas enzymes like Cas9 and Cas12a, are of great interest for genome editing. The specificity of these DNA nucleases is determined by RNA guides, providing great targeting adaptability. Besides this general method of programmable DNA cleavage, these nucleases have different biochemical characteristics, that can be exploited for different applications. Although Cas nucleases are highly promising, some room for improvement remains. New developments and discoveries like base editing, prime editing, and CRISPR-associated transposons might address some of these challenges.

## Introduction

**Figure 1.**
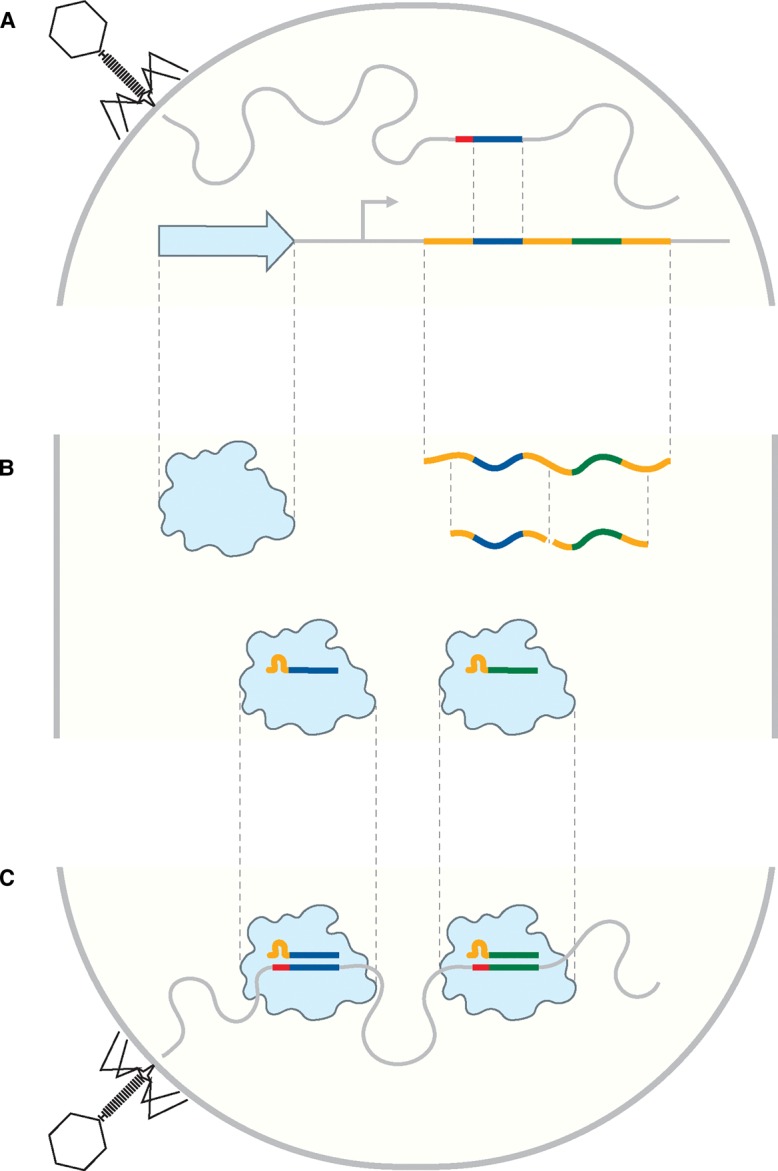
CRISPR–Cas adaptive immunity. Schematic overview of the steps involved in adaptive immunity as provided by CRISPR–Cas. (**A**) Adaptation stage, featuring the introduction of viral DNA/RNA (curved gray line) with protospacer 1 (dark blue) and an adjacent PAM (red). Protospacer 1 is incorporated in the bacterial genome as spacer 1 (dark blue) flanked by two repeats (orange) in the CRISPR array alongside spacer 2 (green). (**B**) Expression stage, wherein a Cas protein (light blue) is produced and pre-crRNA is transcribed and processed into mature crRNAs, which subsequently form functional RNP complexes with the Cas protein. (**C**) Interference stage, where a second infection is targeted for degradation by crRNA-guided Cas proteins, binding to matching protospacers adjacent to PAMs.

Horizontal gene transfer plays a crucial role in the evolution of prokaryotes [1–4]. Invading genetic material may give fitness advantages to recipient cells, but also may pose a substantial risk. Therefore, bacteria and archaea have evolved a range of mechanisms to protect themselves from foreign nucleic acids [5]. One such defense mechanism is the CRISPR–Cas system (clustered regularly interspaced short palindromic repeats and CRISPR-associated). CRISPR–Cas has been shown to provide adaptive immunity against mobile genetic elements (e.g. viruses) in three stages: adaptation, expression, and interference (Figure 1) [6,7].

In the adaptation stage (Figure 1A), a complex of Cas1 and Cas2 (and sometimes Cas4) acquires nucleotide stretches called protospacers. Protospacers are selected based on the presence of a protospacer adjacent motif (PAM) and are preferentially derived from foreign DNA or RNA [8–12]. Acquired protospacers are incorporated as spacers in the CRISPR array on the host chromosome, where they are flanked by repeats [13]. Spacer acquisition has been demonstrated to also involve host proteins as well as CRISPR interference itself [14–19].

During the expression stage (Figure 1B), the CRISPR array is transcribed into a long precursor CRISPR RNA (pre-crRNA) that is subsequently processed into small CRISPR RNAs (crRNAs), each containing a single spacer flanked by parts of the repeat. Next, Cas proteins and the mature crRNA (also referred to as the guide RNA) assemble to form a ribonucleoprotein (RNP) complex.

In the interference stage (Figure 1C), RNPs recognize PAMs and interrogate adjacent sequences for complementary to the crRNA. The RNPs then trigger degradation of the foreign DNA or RNA, either by nuclease activity of the RNP itself or through recruitment of another nuclease, for instance, the helicase/nuclease Cas3 [20]. CRISPR–Cas systems are classified in Class 1 (type I, III, IV) and Class 2 (type II, V, VI) [21]. Class 1 systems use multi-subunit Cas complexes (consisting of multiple different Cas proteins) for interference. In contrast, Class 2 systems are characterized by their single, multi-domain protein nucleases.

## Genome editing

Among applications of CRISPR–Cas, genome editing has attracted the most attention by far. Eukaryotic genome editing uses one of two major DNA double-stranded break (DSB) repair pathways: non-homologous end-joining (NHEJ) or homologous recombination (HR) [22]. NHEJ resolves DSBs in the DNA by ligating both ends, often resulting in small insertions or deletions (indels) [23]. In the case of targeted protein-coding genes, indels are likely to cause frameshifts, effectively knocking out these genes. HR, on the other hand, requires a homologous repair template (ssDNA or dsDNA), which is used for more precise DSB repair [22]. For genome editing, one can supply an artificial repair template with the desired sequence, flanked by sequences homologous to the targeted locus. HR will then incorporate the repair template to repair the DSB, thereby introducing the designed edit. Due to these differences in resolving DSBs, NHEJ is useful for knocking out existing genes, and HR can be used for precise insertions or replacement of DNA. Whereas eukaryotic cells possess both HR and NHEJ repair systems, the HR system is highly cell cycle dependent, being mostly active during S-phase [24]. DNA-cleaving Cas enzymes can be used to create DSBs in desired locations, making them useful in directing genome editing for eukaryotes. In prokaryotes, Cas nucleases are not used to direct DNA repair, but rather to cleave the original DNA sequence. These DSBs kill cells with the original DNA sequence, effectively counter selecting unedited cells and enriching for edited cells.

Where most other DNA nucleases rely on protein–DNA binding for target recognition, Cas nucleases use crRNA–DNA complementarity to determine specificity. New crRNAs can be designed and produced rapidly, providing superior versatility for target specificity of Cas nucleases. In this review, we focus on Class 2 Cas nucleases like Cas9 and Cas12a because these combine targeting versatility with the simplicity of single protein nucleases, making them the preferred tools for genome editing.

## Cas9

Because of its early discovery, the most frequently used Cas protein is the type II nuclease Cas9. The characterization of Cas9 from *Streptococcus pyogenes* (SpCas9) highlighted the potential of CRISPR–Cas for genome editing applications [25,26]. Since then, Cas9 has been used for applications in organisms from all domains of life, ranging from improving microbial cell factories and crop plants to clinical trials aimed at repairing genetic disorders in human patients [27–36].

**Figure 2.**
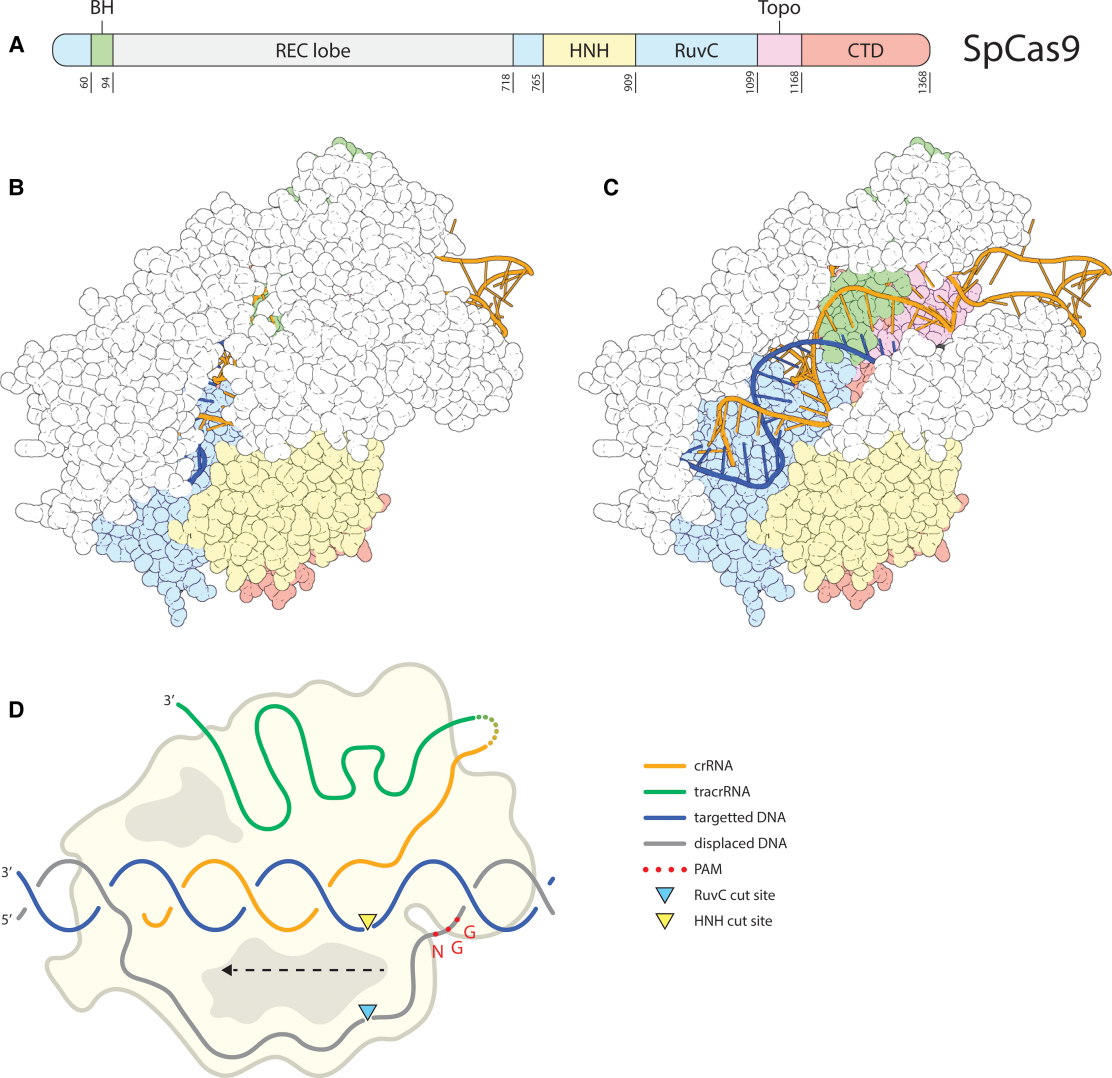
SpCas9. (**A**) The layout of the SpCas9 protein from C- (left) to N-terminus (right). Featuring the REC lobe (gray) and the domains of the NUC lobe: RuvC (light blue), BH (green), HNH (yellow), Topo-homology (Topo, pink), CTD (C-terminal domain, red). The last residue position for each domain is given below the domains. (**B**) Full structure (PDB: 4UN3 [46]) of SpCas9 in complex with sgRNA (orange), target DNA strand (dark blue), and displaced DNA strand (black, but barely visible here). Protein domains are colored in accordance with (**A**). Visualized using PyMOL [109]. (**C**) Same as B with some residues from the REC lobe removed to allow a view of the sgRNA and the target DNA strand. (**D**) Schematic overview of the R-loop formed by Cas9 RNP. DNA strands are colored in accordance with (**B**). The crRNA (orange) and tracrRNA (green) are shown connected by a linker (dashed lines between them) to form a sgRNA. The direction of R-loop formation is indicated with a dashed arrow. Approximate cleavage positions are represented by colored triangles.

Cas9 is a single, multi-domain DNA nuclease that is guided by RNA [25]. The Cas9 protein can be divided into the recognition (REC) lobe and the nuclease (NUC) lobe [37] (Figure 2A). The NUC lobe consists of a PAM-interacting (PI) domain and two nuclease domains: HNH and RuvC. The archetype SpCas9 protein is a polypeptide of 1368 amino acids, but shorter variants from other organisms have also been described [38–40].

In type II systems, CRISPRs are transcribed as a single pre-crRNA. The maturation of the guides requires a transactivating crRNA (tracrRNA), which partially base pairs to the repeat regions of the pre-crRNA [41]. In the presence of Cas9, these partial dsRNA fragments are processed by the non-Cas ribonuclease RNase III [41]. To circumvent this processing step, the mature tracrRNA and crRNA can be fused by a short synthetic linker, and expressed as a single-guide RNA (sgRNA) that binds Cas9 to form a functional RNP complex [25].

The resulting RNP complex surveys the cells for a complementary target sequence. Single-molecule analysis revealed that the Cas9 RNP complex encounters DNA stretches through three-dimensional diffusion [42]. In the absence of a PAM, these RNP–DNA interactions are very short-lived [42]. If the RNP encounters a PAM, however, binding lasts substantially longer [42]. The PAM can, therefore, be regarded as an initial quality control that Cas9 uses in its surveillance for DNA targets and might allow the nuclease to find target DNA sequences more efficiently. SpCas9 recognizes its PAM (5′-NGG), directly downstream of the target sequence (Figure 2D) [25]. As the 5′-NGG motif occurs quite often, the PAM is generally not limiting Cas9 applications, unless a very precise cleavage position is desired. It should be noted that PAM recognition by Cas9 can be either strict or very relaxed in different Cas9 orthologs [39,43–45].

PAM binding by Cas9 enables local separation of the DNA strands directly upstream of the PAM, allowing interrogation of the target sequence for base pairing with the crRNA [42,46]. Successful base-pairing interactions between the interrogated target DNA strand and the crRNA extend the melting of the DNA strands [42]. The DNA strand displacement proceeds towards the PAM-distal region, eventually resulting in a complete R-loop configuration [42] (Figure 2B–D). Because of the direction (PAM proximal to distal), PAM-proximal matching between crRNA and the target DNA are disproportionately important for RNP binding [42,47]. This important PAM-proximal stretch is referred to as the seed region; in case of mismatches between this seed and the target strand, R-loop formation, and subsequent cleavage are generally aborted [25,42,47]. A certain level of mismatches in the PAM-distal target sequence are tolerated, which is advantageous for its natural role in defense against viruses with high mutation rates. However, in the case of genome editing, this tolerance can result in undesired binding/cleavage of imperfectly matching DNA sequences, referred to as off-targets (see below) [48,49]. More specifically, efficient DNA binding by Cas9 requires at least nine continuous matches between crRNA and target sequence [47]. Sufficient base pairing between crRNA and the DNA target strand stabilizes the R-loop, which is further stabilized by the interaction between the displaced DNA strand and charged residues of the Cas9 protein [37,50]. If the R-loop extends to the most PAM-distal bases, a conformational change is induced that poises the HNH domain in the NUC lobe for cleavage [51].

The HNH domain then cleaves the target strand, which consistently creates a break between the 3rd and 4th nucleotides upstream of the PAM [25,52]. The conformational change in the HNH domain is also required for nuclease activity of the RuvC domain which cleaves the displaced strand, generally between either the 3rd and 4th or between the 4th and 5th bases upstream of the PAM [25,53,52]. Collectively, this results in a DSB with blunt ends or a short overhang. Notably, cleavage by Cas9 falls within the seed region of the target sequence [25]. If the dsDNA break is repaired through NHEJ, the resulting insertions or deletions will lead to disruption of the seed region, and hence the altered target sequence is no longer cleaved.

After cleavage, Cas9 remains stably bound to both ends of the protospacer. While the targeted strand is in heteroduplex with the crRNA, the cleaved ends of the displaced strand are less structured and might engage the RuvC active site repeatedly. This would explain the observed time-dependent exonuclease activity on the displaced strand after initial cleavage [52].

## Cas12a

Although Cas9 has proven to be a powerful tool for genome editing, other Cas nucleases may be interesting alternatives. The type V nuclease Cas12a (previously called Cpf1) is a good example of this. Despite having been characterized several years later than Cas9, Cas12a offers some advantages that may favor the latter enzyme for specific applications. One of the most used variants of Cas12a is the enzyme from *Acidaminococcus sp. BV3L6* (AsCas12a). Like Cas9, Cas12a has by now been applied in a wide range of species including bacteria, yeast, plants, and in human cells [54–58].

**Figure 3.**
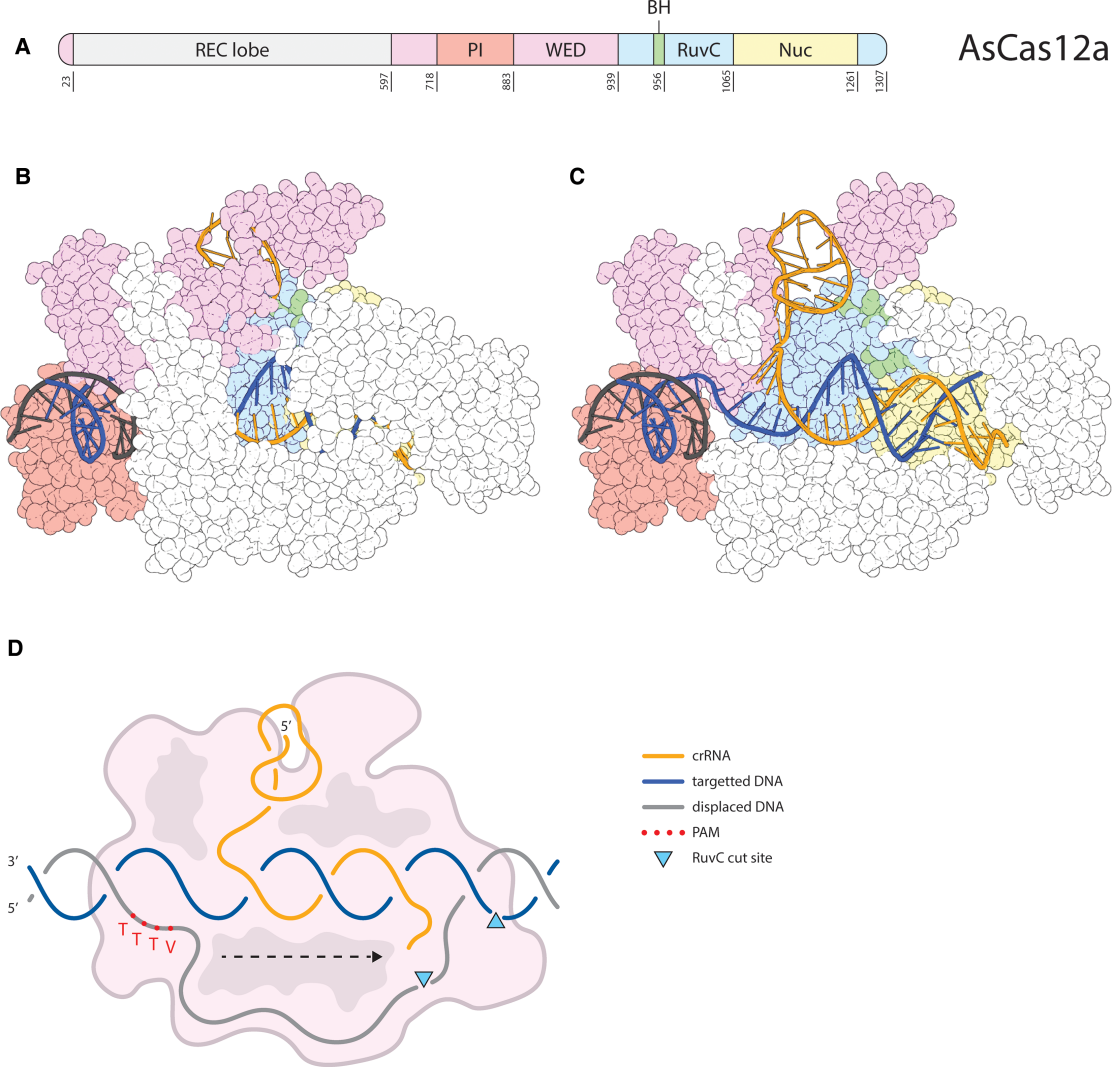
AsCas12a. (**A**) The layout of the AsCas12a protein from C- (left) to N-terminus (right). Featuring the REC lobe (gray) and the domains of the NUC lobe: WED (pink), PI (red), RuvC (light blue), BH (green), Nuc (yellow). The last residue position for each domain is given below the domains. (**B**) Full structure (PDB: 5B43 [60]) of AsCas12a in complex with crRNA (orange), target DNA strand (dark blue), and displaced DNA strand (black). Protein domains are colored in accordance with (**A**). Visualized using PyMOL [109]. (**C**) Same as B with some residues from the REC lobe and WED domain removed to allow a view of the sgRNA and DNA. (**D**) Schematic overview of the R-loop formed by Cas12a RNP. DNA and RNA strands are colored in accordance with (**B**). The direction of R-loop formation is indicated with a dashed arrow. Approximate RuvC cleavage positions in the DNA are represented by (light blue) triangles, but both strands are cleaved by a single catalytic site [62].

As a Class 2 nuclease, Cas12a is a single multi-domain protein that induces RNA-guided dsDNA cleavage [59]. Like Cas9, the Cas12a structure can be subdivided in a REC and a NUC lobe [60,61] (Figure 3A). The NUC lobe of Cas12a lacks an HNH domain, but does contain a RuvC domain, for DNA cleavage [60]. In addition, the NUC lobe features a Wedge (WED) domain and a ‘Nuclease’ (Nuc) domain, which turned out not to cleave DNA [62]. AsCas12a is 1307 amino acids long, making it almost as large as SpCas9.

Unlike Cas9, Cas12a does not require a tracrRNA (nor RNase III) for pre-crRNA processing [59]. However, other type V nucleases (e.g. Cas12b) do need tracrRNAs [63]. Cas12a possesses a ribonuclease site in the WED domain, that allows for autonomous processing of pre-crRNA to mature crRNAs [64]. Cas12a crRNAs are substantially shorter than the Cas9 guide RNA (sgRNA or crRNA + tracrRNA). Collectively, this allows compact Cas12a CRISPR arrays which can be used to target multiple sites simultaneously [65]. Once bound to the Cas12a protein, the repeat-derived part of the crRNA adopts a 5′ pseudoknot structure [66]. Within the spacer-derived part of the crRNA, the PAM-proximal (seed) region is pre-ordered by interactions with the protein, exposing these nucleotides for base-pairing interactions with the DNA target strand [62].

In contrast with Cas9, the Cas12a RNP seems to follow the DNA through intermittent contact during its survey for protospacers [67], although it would be best to compare DNA surveillance by both nucleases in a single study. Cas12a enzymes recognize target sequence-upstream T-rich PAMs (5′-TTTV for AsCas12a) on the displaced strand [59] (Figure 3D). The T-rich PAM of Cas12a expands the targeting space that Cas9 offers.

PAM binding by Cas12a causes local melting of the target sequence DNA directly downstream of the PAM [60–62]. This allows the interrogation of the downstream sequence for complementarity to the crRNA. The crRNA base pairs to the DNA target strand, further melting the DNA, extending the heteroduplex towards the PAM-distal end, eventually forming a full R-loop [67] (Figure 3B–D). This directionality results in a PAM-proximal seed region where mismatches are more deleterious to binding and cleavage [68]. However, compared with Cas9, Cas12a requires more matching bases between crRNA and target DNA for stable binding [47,68,69]. Especially, PAM-distal mismatches are much less tolerated by Cas12a than by Cas9 [69]. Cas12a thus has a less pronounced seed region and provides more specificity, as observed *in vivo* [70,69]. Only when target DNA and crRNA are sufficiently complementary, the Cas12a RNP forms a stable R-loop structure, enabling DNA cleavage [67,68].

Unlike Cas9, Cas12a likely uses a single RuvC nuclease domain to cleave both strands of DNA [62]. The Cas12a RuvC domain is thought to first cleave the displaced strand, generally between the 16th and 17th nucleotides downstream from the PAM, then proceeding to cleave the target strand between the 23rd and 24th nucleotides [67,69,71]. However, the initial cleavage sites have been found to vary slightly and subsequent trimming (see below) is thought to take place as well [69]. The Nuc domain seems to be involved in orienting the target strand DNA close to the active site that resides in the RuvC domain [62]. This is supported by the observation that a mutation in the Nuc domain (R1226A for AsCas12a) prevents cleavage of the target strand, effectively converting Cas12a into a nickase [60].

Cas12a thus induces PAM-distal cleavage with a 5′ overhang of variable length. It is worth noting that Cas12a cleaves outside the seed region, while Cas9 cleaves within its seed region. Because of this, Zetsche et al. [59] suggested that DNA cleavage by Cas12a could tolerate small indels created by NHEJ, resulting in multiple rounds of DSB formation, as such enhancing the chance of HR to occur. However, given the high observed *in vivo* specificity of Cas12a, this now seems unlikely [70].

After cleavage, the Cas12a RNP remains bound to the PAM-proximal cleavage product [68]. This allows the displaced strand to be trimmed likely due to subsequent interactions with the RuvC catalytic site [69]. In contrast with the relatively stable interaction of Cas9 with its cleavage products, the Cas12a PAM-distal cleavage product is readily released after cleavage [42,47,68]. This release of the PAM-distal DNA might allow ssDNA molecules to interact with the still active RuvC domain, possibly explaining the observed non-specific cleavage activity on ssDNA by Cas12a after cleavage of an initial specific dsDNA target [72,73]. This conditional, collateral ssDNA activity is unlikely to be problematic as DNA hardly occurs in a single-stranded state *in vivo*. Indeed, Cas12a has been found to be less toxic than Cas9 in some contexts [54,74].

## Challenges for genome editing

Although both Cas9 and Cas12a are highly promising tools, some room for improvement remains for their optimal performance in genome editing. One main constraint of Cas nucleases is that the targeting space is limited by the requirement for the presence of a properly positioned PAM. To overcome this limitation, rational engineering and/or random mutagenesis have been used to produce Cas9 and Cas12a enzymes with altered, or less stringent PAM recognition [46,75–78]. It is tempting to propose efforts towards a hypothetical PAM-less Cas nuclease. However, the PAM recognition is generally coupled to the initial opening of the dsDNA target. In addition, more permissive Cas nucleases would spend more time on non-target sequences [42]. Because of this, there might be a strict trade-off between PAM range and on-target activity.

In contrast with Cas nucleases, prokaryotic Argonaute proteins are a class of guided nucleases that do not require a PAM for their activity. Because currently characterized prokaryotic Argonaute proteins do not trigger DNA unwinding, their dsDNA cleavage activity is far lower than for Cas9 and Cas12a [79,80]. Hence, it will be a major challenge to develop an efficient PAM-independent Cas nuclease.

An alternative to PAM-less nucleases is to work towards a comprehensive ‘toolbox’ of Cas nucleases that each recognize a different PAM. A Cas nuclease with an appropriate PAM could then be chosen based on the desired target site. The current targeting range is most limiting for applications where the exact location of cleavage is critical, including single nucleotide engineering by base editing (see below) [75].

The presence of a PAM does not always guarantee efficient protospacer cleavage. In addition to the PAM, the nature of the crRNA/protospacer sequence has been found to strongly affect the cleavage efficiency of Cas nucleases [48,49]. Apart from target accessibility related to chromatin structure, the variable parts of the (pre-)crRNA sequences may result in the formation of inappropriate secondary structures. Mis-folding of these guides will affect the formation of functional RNP complexes, resulting in decreased editing efficiency.

However, high cleavage efficacy does not always result in efficient eukaryotic genome editing. HR efficiency in eukaryotic cells varies substantially depending on the cell type and on the cell cycle phase. In addition, the NHEJ system may outcompete the HR system in repairing DSBs, which might lead to low levels of the desired HR. In conclusion, for precise genome editing, HR efficiency is a major bottleneck, rather than DSB formation by Cas nucleases. Hence, apart from strategies to improve HR, HR-independent strategies (see below) may be promising alternatives [81].

Possibly the most important challenge for applications of Cas nucleases is to solve the problem of off-target cleavage. Indeed sequences partially complementary to the crRNA have been shown to be cleaved at relevant rates [48,82]. For human therapeutics, it is critical that these off-targets are reduced to the bare minimum to enable safe genome editing. Compared with Cas9, Cas12a is substantially more specific due to its decreased tolerance for mismatches during DNA/RNA heteroduplex formation, at least *in vivo* [70]. Characterization of more natural Cas nucleases might reveal even more specific enzymes. In addition, rational engineering combined with laboratory evolution has been used to successfully generate several Cas9 variants with substantially increased specificity [50,83–88].

## Beyond DNA cleavage by Cas9 and Cas12a

Several exciting developments (Table 1) have recently been established in the field of Class II CRISPR nucleases. These developments further highlight the potential of the programmable DNA-specificity of Cas nucleases. Besides Cas9 and Cas12a, other Cas nucleases may prove useful genome editing tools, including Cas12b and Cas12e nucleases for which genome editing has already been demonstrated [63,89]. In addition, a designed variant of a Class 1 complex (Cascade fused to a FokI nuclease domain) has recently been described as an alternative genome editing system [90].

Active nucleases like Cas9 and Cas12a can be converted into nickases or be rendered catalytically inactive by mutating conserved residues in the catalytic sites [25,91,59,60]. Binding of such catalytically inactive Cas nucleases can be used to inhibit gene transcription (CRISPR inhibition, CRISPRi), e.g. by blocking a promoter to prevent binding of RNA polymerase [91–93]. On the other hand, fusions of a Cas nuclease to the RNA polymerase omega subunit can be used to enhance the recruitment of RNA polymerase, promoting expression (CRISPR activation, CRISPRa) of downstream genes [92–94]. This type of targeted gene regulation can be used to study gene functions and is a versatile tool in the field of synthetic biology.

Nickase and catalytically inactive versions of Cas nucleases also offer a tantalizing alternative to the DSB-induced genome editing. The programmable specificity of Cas nucleases can be used to direct fused cytidine or adenine deaminase enzymes to specific DNA stretches. Such base editing has so far been reported for conversions of C*G pairs to T*A (using cytidine deaminase fused Cas9 or Cas12a), or A*T to G*C (using an evolved and engineered DNA adenine deaminase fused Cas9) [95–98].

Compared with DSB-repair-based genome editing, base editing offers major advantages. Most importantly, base conversions do not rely on HR, NHEJ or other mechanisms of DSB repair. Therefore, base editing could theoretically reach higher efficiencies as it is less dependent on cell type and cell cycle phase. In addition, because no DSB is introduced with base editing, it is unlikely to result in major chromosome rearrangements [99]. Recently, cytosine base editing (but not adenine base editing) was found to induce substantial off-target base substitutions on DNA, possibly due to protospacer independent base conversions [100,101]. In addition, both types of base editors were found to convert off-target bases in cellular RNA, although such RNA activity could be reduced through protein engineering [102,103]. These studies highlight the importance of genome/transcriptome wide off-target analysis on single cell-derived DNA. Another challenge for base editing is the requirement for precise targeting, which is often limited by PAM availability (see above). Lastly, it is currently difficult to target a single base position for conversion, instead, current base editors may convert any appropriate base within a certain window (although these windows can be very narrow). Whereas this is no problem for gene disruption, it may be a major hurdle for precision gene therapy. Future optimization of base editors is required to address these challenges.

The recent development of prime editing potentially offers a much more versatile method for DSB-independent genome editing [104]. Prime editing uses an extended version of the guide RNA in conjunction with a nickase version of Cas9 fused to reverse transcriptase. The RNA guides the Cas9 fusion to the complementary target on the genome, where Cas9 nicks the displaced strand. The reverse transcriptase then uses the extra part of the guide RNA as a primer, incorporating the guide-encoded edit in the DNA of the displaced strand. Prime editing enables small insertions, deletions, and replacements to be made independent from NHEJ or HR.

Another exciting development is the application of recently discovered CRISPR-associated transposons for both Class I and Class II CRISPR systems [105–108]. These transposons rely on RNA-guided Cas enzymes to direct transposition to positions downstream from protospacers. The involved Cas proteins do not introduce DSBs, but rather guide the transposon proteins [107]. This targeted transposase activity has already been used to integrate large stretches of DNA in the genome of *Escherichia coli* at high efficiency [107,108]. Although it requires transposon proteins in addition to the Cas nuclease protein(s), this method provides a promising alternative to HR-based genomic insertions. The next challenge will be to successfully use these systems for programmable integrations in mammalian cells.

**Table 1:** Genome editing strategies

Strategy	Edit type	Features
NHEJ	Small indels [23]	• Triggered by DSBs • No control over indels • Useful for knock-outs
HR	Replacements [22]	• Triggered by DSBs • Cell-cycle dependent • Requires repair template
Counter selection	Selection only	• Relies on HR • Enriches edited cells • Useful for prokaryotes
Base editing [95–97]	Base substitutions	• Independent of DNA repair • Single base substitutions • Sensitive to off-targets [100,101]
Prime editing [104]	Replacements	• Independent of DNA repair • Versatile for small edits • Control over exact edit
Transposition [107,108]	Insertions	• Requires transposon proteins • Large insertions possible • So far only in prokaryotes

Overview of the six main strategies for genome editing based on CRISPR–Cas, listing the type of edit they introduce and three main features per strategy.

## Perspectives

- The CRISPR–Cas defense system of prokaryotes holds great promise for genome editing in a broad spectrum of organisms, across the entire tree of life. In fact, Cas nuclease-based cleavage and binding of DNA have already proven extremely useful in fundamental research and in microbial and plant biotechnology.
- Apart from the initial biochemical and genetic analyses, a range of high-resolution structures and single-molecule studies have shed light on the molecular mechanism of DNA cleavage by Class 2 Cas nucleases. It has also become apparent that there is room for improving targeting space, specificity, and efficiency for genome editing.
- Engineering by rational design and/or laboratory evolution may result in improved variants of the current set of Cas nucleases, potentially addressing recent challenges. Moving forward, we expect more developments in engineering Cas nucleases for applications like base editing and prime editing. Finally, it is anticipated that future research will reveal CRISPR systems with new Cas proteins, potentially offering new functions as exemplified by the newly discovered role in RNA-guided transposition.

## Abbreviations

AsCas12a: *Acidaminococcus sp. BV3L6* Cas12a
BH domain: bridge-helix domain
Cas: CRISPR-associated
CRISPR: clustered regularly interspaced short palindromic repeats
CRISPRa: CRISPR activation
CRISPRi: CRISPR inhibition
crRNA: CRISPR RNA
CTD: C-terminal domain
DSB: double-stranded break
HR: homologous recombination
Indels: insertions and deletions
NHEJ: non-homologous end-joining
Nuc domain: ‘Nuclease’ domain
NUC lobe: nuclease lobe
PAM: protospacer adjacent motif
PI domain: PAM-interacting domain
pre-crRNA: precursor CRISPR RNA
REC lobe: recognition lobe
RNP: ribonucleoprotein
sgRNA: single-guide RNA
SpCas9: *Streptococcus pyogenes* Cas9
Topo domain: Topo-homology domain
tracrRNA: transactivating crRNA
WED domain: Wedge domain

## References

[1] de la Cruz F. and Davies J. (2000) Horizontal gene transfer and the origin of species: lessons from bacteria. Trends Microbiol. 8, 128–133 10.1016/S0966-842X(00)01703-0 10707066

[2] Gogarten J.P., Doolittle W.F. and Lawrence J.G. (2002) Prokaryotic evolution in light of gene transfer. Mol. Biol. Evol. 19, 2226–2238 10.1093/oxfordjournals.molbev.a004046 12446813

[3] Chibani-Chennoufi S., Bruttin A., Dillmann M.-L. and Brüssow H. (2004) Phage-host interaction: an ecological perspective. J. Bacteriol. 186, 3677–3686 10.1128/JB.186.12.3677-3686.2004 15175280 PMC419959

[4] Koonin E.V. and Wolf Y.I. (2008) Genomics of bacteria and archaea: the emerging dynamic view of the prokaryotic world. Nucleic Acids Res. 36, 6688–6719 10.1093/nar/gkn668 18948295 PMC2588523

[5] Koonin E.V., Makarova K.S. and Wolf Y.I. (2017) Evolutionary genomics of defense systems in archaea and bacteria. Annu. Rev. Microbiol. 71, 233–261 10.1146/annurev-micro-090816-093830 28657885 PMC5898197

[6] Barrangou R., Fremaux C., Deveau H., Richards M., Boyaval P., Moineau S. et al. (2007) CRISPR provides acquired resistance against viruses in prokaryotes. Science 315, 1709–1712 10.1126/science.1138140 17379808

[7] van der Oost J., Jore M.M., Westra E.R., Lundgren M. and Brouns S.J.J. (2009) CRISPR-based adaptive and heritable immunity in prokaryotes. Trends Biochem. Sci. 34, 401–407 10.1016/j.tibs.2009.05.002 19646880

[8] Nuñez J.K., Kranzusch P.J., Noeske J., Wright A.V., Davies C.W. and Doudna J.A. (2014) Cas1–Cas2 complex formation mediates spacer acquisition during CRISPR–Cas adaptive immunity. Nat. Struct. Mol. Biol. 21, 528–534 10.1038/nsmb.2820 24793649 PMC4075942

[9] Bolotin A., Quinquis B., Sorokin A. and Ehrlich S.D. (2005) Clustered regularly interspaced short palindrome repeats (CRISPRs) have spacers of extrachromosomal origin. Microbiology 151, 2551–2561 10.1099/mic.0.28048-0 16079334

[10] Horvath P., Romero D.A., Coûté-Monvoisin A.-C., Richards M., Deveau H., Moineau S. et al. (2008) Diversity, activity, and evolution of CRISPR Loci in *Streptococcus thermophilus*. J. Bacteriol. 190, 1401–1412 10.1128/JB.01415-07 18065539 PMC2238196

[11] Mojica F.J.M., Díez-Villaseñor C., García-Martínez J. and Almendros C. (2009) Short motif sequences determine the targets of the prokaryotic CRISPR defence system. Microbiology 155, 733–740 10.1099/mic.0.023960-0 19246744

[12] Marraffini L.A. and Sontheimer E.J. (2010) Self versus non-self discrimination during CRISPR RNA-directed immunity. Nature 463, 568–571 10.1038/nature08703 20072129 PMC2813891

[13] Yosef I., Goren M.G. and Qimron U. (2012) Proteins and DNA elements essential for the CRISPR adaptation process in *Escherichia coli*. Nucleic Acids Res. 40, 5569–5576 10.1093/nar/gks216 22402487 PMC3384332

[14] Levy A., Goren M.G., Yosef I., Auster O., Manor M., Amitai G. et al. (2015) CRISPR adaptation biases explain preference for acquisition of foreign DNA. Nature 520, 505–510 10.1038/nature14302 25874675 PMC4561520

[15] Radovčić M., Killelea T., Savitskaya E., Wettstein L., Bolt E.L. and Ivančić-Baće I. (2018) CRISPR–cas adaptation in *Escherichia coli* requires RecBCD helicase but not nuclease activity, is independent of homologous recombination, and is antagonized by 5′ ssDNA exonucleases. Nucleic Acids Res. 46, 10173–10183 10.1093/nar/gky799 30189098 PMC6212769

[16] Swarts D.C., Mosterd C., van Passel M.W.J. and Brouns S.J.J. (2012) CRISPR interference directs strand specific spacer acquisition. PLoS ONE 7, e35888 10.1371/journal.pone.0035888 22558257 PMC3338789

[17] Datsenko K.A., Pougach K., Tikhonov A., Wanner B.L., Severinov K. and Semenova E. (2012) Molecular memory of prior infections activates the CRISPR/Cas adaptive bacterial immunity system. Nat. Commun. 3, 1–7 10.1038/ncomms1937 22781758

[18] Staals R.H.J., Jackson S.A., Biswas A., Brouns S.J.J., Brown C.M. and Fineran P.C. (2016) Interference-driven spacer acquisition is dominant over naive and primed adaptation in a native CRISPR–Cas system. Nat. Commun. 7, 12853 10.1038/ncomms12853 27694798 PMC5059440

[19] Künne T., Kieper S.N., Bannenberg J.W., Vogel A.I.M., Miellet W.R., Klein M. et al. (2016) Cas3-derived target DNA degradation fragments fuel primed CRISPR adaptation. Mol. Cell. 63, 852–864 10.1016/j.molcel.2016.07.011 27546790

[20] Marraffini L.A. (2015) CRISPR–Cas immunity in prokaryotes. Nature 526, 55–61 10.1038/nature15386 26432244

[21] Makarova K.S., Wolf Y.I., Alkhnbashi O.S., Costa F., Shah S.A., Saunders S.J. et al. (2015) An updated evolutionary classification of CRISPR–Cas systems. Nat. Rev. Microbiol. 13, 722–736 10.1038/nrmicro3569 26411297 PMC5426118

[22] Scully R., Panday A., Elango R. and Willis N.A. (2019) DNA double-strand break repair-pathway choice in somatic mammalian cells. Nat. Rev. Mol. Cell Biol. 20, 698–714 10.1038/s41580-019-0152-0 31263220 PMC7315405

[23] Ceccaldi R., Rondinelli B. and D'Andrea A.D. (2016) Repair pathway choices and consequences at the double-strand break. Trends Cell Biol. 26, 52–64 10.1016/j.tcb.2015.07.009 26437586 PMC4862604

[24] Karanam K., Kafri R., Loewer A. and Lahav G. (2012) Quantitative live cell imaging reveals a gradual shift between DNA repair mechanisms and a maximal use of HR in mid S phase. Mol. Cell. 47, 320–329 10.1016/j.molcel.2012.05.052 22841003 PMC3494418

[25] Jinek M., Chylinski K., Fonfara I., Hauer M., Doudna J.A. and Charpentier E. (2012) A programmable dual-RNA-guided DNA endonuclease in adaptive bacterial immunity. Science 337, 816–821 10.1126/science.1225829 22745249 PMC6286148

[26] Gasiunas G., Barrangou R., Horvath P. and Siksnys V. (2012) Cas9-crRNA ribonucleoprotein complex mediates specific DNA cleavage for adaptive immunity in bacteria. Proc. Natl Acad. Sci. U.S.A. 109, E2579–E2586 10.1073/pnas.1208507109 22949671 PMC3465414

[27] Xie K. and Yang Y. (2013) RNA-guided genome editing in plants using a CRISPR–Cas system. Mol. Plant. 6, 1975–1983 10.1093/mp/sst119 23956122

[28] Wang H., Yang H., Shivalila C.S., Dawlaty M.M., Cheng A.W., Zhang F. et al. (2013) One-step generation of mice carrying mutations in multiple genes by CRISPR/Cas-mediated genome engineering. Cell 153, 910–918 10.1016/j.cell.2013.04.025 23643243 PMC3969854

[29] Shan Q., Wang Y., Li J., Zhang Y., Chen K., Liang Z. et al. (2013) Targeted genome modification of crop plants using a CRISPR–Cas system. Nat. Biotechnol. 31, 686–688 10.1038/nbt.2650 23929338

[30] Nekrasov V., Staskawicz B., Weigel D., Jones J.D.G. and Kamoun S. (2013) Targeted mutagenesis in the model plant *Nicotiana benthamiana* using Cas9 RNA-guided endonuclease. Nat. Biotechnol. 31, 691–693 10.1038/nbt.2655 23929340

[31] Mali P., Yang L., Esvelt K.M., Aach J., Guell M., DiCarlo J.E. et al. (2013) RNA-guided human genome engineering via Cas9. Science 339, 823–826 10.1126/science.1232033 23287722 PMC3712628

[32] Li J.-F., Norville J.E., Aach J., McCormack M., Zhang D., Bush J. et al. (2013) Multiplex and homologous recombination–mediated genome editing in *Arabidopsis* and *Nicotiana benthamiana* using guide RNA and Cas9. Nat. Biotechnol. 31, 688–691 10.1038/nbt.2654 23929339 PMC4078740

[33] Hwang W.Y., Fu Y., Reyon D., Maeder M.L., Tsai S.Q., Sander J.D. et al. (2013) Efficient genome editing in zebrafish using a CRISPR–Cas system. Nat. Biotechnol. 31, 227–229 10.1038/nbt.2501 23360964 PMC3686313

[34] Friedland A.E., Tzur Y.B., Esvelt K.M., Colaiácovo M.P., Church G.M. and Calarco J.A. (2013) Heritable genome editing in *C. elegans* via a CRISPR-Cas9 system. Nat. Methods 10, 741–743 10.1038/nmeth.2532 23817069 PMC3822328

[35] Cong L., Ran F.A., Cox D., Lin S., Barretto R., Habib N. et al. (2013) Multiplex genome engineering using CRISPR/Cas systems. Science 339, 819–823 10.1126/science.1231143 23287718 PMC3795411

[36] Bassett A.R., Tibbit C., Ponting C.P. and Liu J.-L. (2013) Highly efficient targeted mutagenesis of *Drosophila* with the CRISPR/Cas9 system. Cell Rep. 4, 220–228 10.1016/j.celrep.2013.06.020 23827738 PMC3714591

[37] Nishimasu H., Ran F.A., Hsu P.D., Konermann S., Shehata S.I., Dohmae N. et al. (2014) Crystal structure of Cas9 in complex with guide RNA and target DNA. Cell 156, 935–949 10.1016/j.cell.2014.02.001 24529477 PMC4139937

[38] Hou Z., Zhang Y., Propson N.E., Howden S.E., Chu L.-F., Sontheimer E.J. et al. (2013) Efficient genome engineering in human pluripotent stem cells using Cas9 from *Neisseria meningitidis*. Proc. Natl Acad. Sci. U.S.A. 110, 15644–15649 10.1073/pnas.1313587110 23940360 PMC3785731

[39] Ran F.A., Cong L., Yan W.X., Scott D.A., Gootenberg J.S., Kriz A.J. et al. (2015) *In vivo* genome editing using *Staphylococcus aureus* Cas9. Nature 520, 186–191 10.1038/nature14299 25830891 PMC4393360

[40] Kim E., Koo T., Park S.W., Kim D., Kim K., Cho H.-Y. et al. (2017) *In vivo* genome editing with a small Cas9 orthologue derived from *Campylobacter jejuni*. Nat. Commun. 8, 14500 10.1038/ncomms14500 28220790 PMC5473640

[41] Deltcheva E., Chylinski K., Sharma C.M., Gonzales K., Chao Y., Pirzada Z.A. et al. (2011) CRISPR RNA maturation by trans-encoded small RNA and host factor RNase III. Nature 471, 602–607 10.1038/nature09886 21455174 PMC3070239

[42] Sternberg S.H., Redding S., Jinek M., Greene E.C. and Doudna J.A. (2014) DNA interrogation by the CRISPR RNA-guided endonuclease Cas9. Nature 507, 62–67 10.1038/nature13011 24476820 PMC4106473

[43] Hirano H., Gootenberg J.S., Horii T., Abudayyeh O.O., Kimura M., Hsu P.D. et al. (2016) Structure and engineering of *Francisella novicida* Cas9. Cell 164, 950–961 10.1016/j.cell.2016.01.039 26875867 PMC4899972

[44] Harrington L.B., Paez-Espino D., Staahl B.T., Chen J.S., Ma E., Kyrpides N.C. et al. (2017) A thermostable Cas9 with increased lifetime in human plasma. Nat. Commun. 8, 1424 10.1038/s41467-017-01408-4 29127284 PMC5681539

[45] Mougiakos I., Mohanraju P., Bosma E.F., Vrouwe V., Finger Bou M., Naduthodi M.I.S. et al. (2017) Characterizing a thermostable Cas9 for bacterial genome editing and silencing. Nat. Commun. 8, 1647 10.1038/s41467-017-01591-4 29162801 PMC5698299

[46] Anders C., Niewoehner O., Duerst A. and Jinek M. (2014) Structural basis of PAM-dependent target DNA recognition by the Cas9 endonuclease. Nature 513, 569–573 10.1038/nature13579 25079318 PMC4176945

[47] Singh D., Sternberg S.H., Fei J., Doudna J.A. and Ha T. (2016) Real-time observation of DNA recognition and rejection by the RNA-guided endonuclease Cas9. Nat. Commun. 7, 12778 10.1038/ncomms12778 27624851 PMC5027287

[48] Hsu P.D., Scott D.A., Weinstein J.A., Ran F.A., Konermann S., Agarwala V. et al. (2013) DNA targeting specificity of RNA-guided Cas9 nucleases. Nat. Biotechnol. 31, 827–832 10.1038/nbt.2647 23873081 PMC3969858

[49] Mandal P.K., Ferreira L.M.R., Collins R., Meissner T.B., Boutwell C.L., Friesen M. et al. (2014) Efficient ablation of genes in human hematopoietic stem and effector cells using CRISPR/Cas9. Cell Stem Cell. 15, 643–652 10.1016/j.stem.2014.10.004 25517468 PMC4269831

[50] Slaymaker I.M., Gao L., Zetsche B., Scott D.A., Yan W.X. and Zhang F. (2016) Rationally engineered Cas9 nucleases with improved specificity. Science 351, 84–88 10.1126/science.aad5227 26628643 PMC4714946

[51] Yang M., Peng S., Sun R., Lin J., Wang N. and Chen C. (2018) The conformational dynamics of Cas9 governing DNA cleavage are revealed by single-molecule FRET. Cell Rep. 22, 372–382 10.1016/j.celrep.2017.12.048 29320734

[52] Stephenson A.A., Raper A.T. and Suo Z. (2018) Bidirectional degradation of DNA cleavage products catalyzed by CRISPR/Cas9. J. Am. Chem. Soc. 140, 3743–3750 10.1021/jacs.7b13050 29461055

[53] Sternberg S.H., LaFrance B., Kaplan M. and Doudna J.A. (2015) Conformational control of DNA target cleavage by CRISPR–Cas9. Nature 527, 110–113 10.1038/nature15544 26524520 PMC4859810

[54] Ungerer J. and Pakrasi H.B. (2016) Cpf1 is a versatile tool for CRISPR genome editing across diverse species of cyanobacteria. Sci. Rep. 6, 39681 10.1038/srep39681 28000776 PMC5175191

[55] Yan M.-Y., Yan H.-Q., Ren G.-X., Zhao J.-P., Guo X.-P. and Sun Y.-C. (2017) CRISPR–Cas12a-assisted recombineering in bacteria. Appl. Environ. Microbiol. 83, e00947-17 10.1128/AEM.00947-17 28646112 PMC5561284

[56] Świat M.A., Dashko S., den Ridder M., Wijsman M., van der Oost J., Daran J.-M. et al. (2017) Fncpf1: a novel and efficient genome editing tool for *Saccharomyces cerevisiae*. Nucleic Acids Res. 45, 12585–12598 10.1093/nar/gkx1007 29106617 PMC5716609

[57] Begemann M.B., Gray B.N., January E., Gordon G.C., He Y., Liu H. et al. (2017) Precise insertion and guided editing of higher plant genomes using Cpf1 CRISPR nucleases. Sci. Rep. 7, 11606 10.1038/s41598-017-11760-6 28912524 PMC5599503

[58] Kim D., Kim J., Hur J.K., Been K.W., Yoon S. and Kim J.-S. (2016) Genome-wide analysis reveals specificities of Cpf1 endonucleases in human cells. Nat. Biotechnol. 34, 863–868 10.1038/nbt.3609 27272384

[59] Zetsche B., Gootenberg J.S., Abudayyeh O.O., Slaymaker I.M., Makarova K.S., Essletzbichler P. et al. (2015) Cpf1 Is a single RNA-guided endonuclease of a class 2 CRISPR–Cas system. Cell 163, 759–771 10.1016/j.cell.2015.09.038 26422227 PMC4638220

[60] Yamano T., Nishimasu H., Zetsche B., Hirano H., Slaymaker I.M., Li Y. et al. (2016) Crystal structure of Cpf1 in complex with guide RNA and target DNA. Cell 165, 949–962 10.1016/j.cell.2016.04.003 27114038 PMC4899970

[61] Gao P., Yang H., Rajashankar K.R., Huang Z. and Patel D.J. (2016) Type V CRISPR–Cas Cpf1 endonuclease employs a unique mechanism for crRNA-mediated target DNA recognition. Cell Res. 26, 901–913 10.1038/cr.2016.88 27444870 PMC4973337

[62] Swarts D.C., van der Oost J. and Jinek M. (2017) Structural basis for guide RNA processing and seed-dependent DNA targeting by CRISPR–Cas12a. Mol. Cell. 66, 221–233.e4 10.1016/j.molcel.2017.03.016 28431230 PMC6879319

[63] Strecker J., Jones S., Koopal B., Schmid-Burgk J., Zetsche B., Gao L. et al. (2019) Engineering of CRISPR–Cas12b for human genome editing. Nat. Commun. 10, 212 10.1038/s41467-018-08224-4 30670702 PMC6342934

[64] Fonfara I., Richter H., Bratovič M., Le Rhun A. and Charpentier E. (2016) The CRISPR-associated DNA-cleaving enzyme Cpf1 also processes precursor CRISPR RNA. Nature 532, 517–521 10.1038/nature17945 27096362

[65] Adiego-Pérez B., Randazzo P., Daran J.M., Verwaal R., Roubos J.A., Daran-Lapujade P. et al. (2019) Multiplex genome editing of microorganisms using CRISPR–Cas. FEMS Microbiol. Lett. 366, fnz086 10.1093/femsle/fnz086 31087001 PMC6522427

[66] Dong D., Ren K., Qiu X., Zheng J., Guo M., Guan X. et al. (2016) The crystal structure of Cpf1 in complex with CRISPR RNA. Nature 532, 522–526 10.1038/nature17944 27096363

[67] Jeon Y., Choi Y.H., Jang Y., Yu J., Goo J., Lee G. et al. (2018) Direct observation of DNA target searching and cleavage by CRISPR–Cas12a. Nat. Commun. 9, 1–11 10.1038/s41467-018-05245-x 30018371 PMC6050341

[68] Singh D., Mallon J., Poddar A., Wang Y., Tippana R., Yang O. et al. (2018) Real-time observation of DNA target interrogation and product release by the RNA-guided endonuclease CRISPR Cpf1 (Cas12a). Proc. Natl Acad. Sci. U.S.A. 115, 5444–5449 10.1073/pnas.1718686115 29735714 PMC6003496

[69] Strohkendl I., Saifuddin F.A., Rybarski J.R., Finkelstein I.J. and Russell R. (2018) Kinetic basis for DNA target specificity of CRISPR–Cas12a. Mol. Cell. 71, 816–824.e3 10.1016/j.molcel.2018.06.043 30078724 PMC6679935

[70] Kleinstiver B.P., Tsai S.Q., Prew M.S., Nguyen N.T., Welch M.M., Lopez J.M. et al. (2016) Genome-wide specificities of CRISPR–Cas Cpf1 nucleases in human cells. Nat. Biotechnol. 34, 869–874 10.1038/nbt.3620 27347757 PMC4980201

[71] Swarts D.C. and Jinek M. (2018) Cas9 versus Cas12a/Cpf1: structure–function comparisons and implications for genome editing. Wiley Interdiscip. Rev. RNA. 9, e1481 10.1002/wrna.1481 29790280

[72] Li S.-Y., Cheng Q.-X., Liu J.-K., Nie X.-Q., Zhao G.-P. and Wang J. (2018) CRISPR–cas12a has both cis - and trans -cleavage activities on single-stranded DNA. Cell Res. 28, 491–493 10.1038/s41422-018-0022-x 29531313 PMC5939048

[73] Chen J.S., Ma E., Harrington L.B., Costa M.D., Tian X., Palefsky J.M. et al. (2018) CRISPR–cas12a target binding unleashes indiscriminate single-stranded DNase activity. Science 360, 436–439 10.1126/science.aar6245 29449511 PMC6628903

[74] Jiang Y., Qian F., Yang J., Liu Y., Dong F., Xu C. et al. (2017) CRISPR–cpf1 assisted genome editing of *Corynebacterium glutamicum*. Nat. Commun. 8, 15179 10.1038/ncomms15179 28469274 PMC5418603

[75] Kleinstiver B.P., Prew M.S., Tsai S.Q., Topkar V.V., Nguyen N.T., Zheng Z. et al. (2015) Engineered CRISPR–Cas9 nucleases with altered PAM specificities. Nature 523, 481–485 10.1038/nature14592 26098369 PMC4540238

[76] Kleinstiver B.P., Prew M.S., Tsai S.Q., Nguyen N.T., Topkar V.V., Zheng Z. et al. (2015) Broadening the targeting range of *Staphylococcus aureus* CRISPR–Cas9 by modifying PAM recognition. Nat. Biotechnol. 33, 1293–1298 10.1038/nbt.3404 26524662 PMC4689141

[77] Gao L., Cox D.B.T., Yan W.X., Manteiga J.C., Schneider M.W., Yamano T. et al. (2017) Engineered Cpf1 variants with altered PAM specificities. Nat. Biotechnol. 35, 789–792 10.1038/nbt.3900 28581492 PMC5548640

[78] Hu J.H., Miller S.M., Geurts M.H., Tang W., Chen L., Sun N. et al. (2018) Evolved Cas9 variants with broad PAM compatibility and high DNA specificity. Nature 556, 57–63 10.1038/nature26155 29512652 PMC5951633

[79] Hegge J.W., Swarts D.C. and Oost van der J. (2018) Prokaryotic Argonaute proteins: novel genome-editing tools? Nat. Rev. Microbiol. 16, 5–11 10.1038/nrmicro.2017.73 28736447

[80] Hegge J.W., Swarts D.C., Chandradoss S.D., Cui T.J., Kneppers J., Jinek M. et al. (2019) DNA-guided DNA cleavage at moderate temperatures by *Clostridium butyricum* Argonaute. Nucleic Acids Res. 47, 5809–5821 10.1093/nar/gkz306 31069393 PMC6582352

[81] Lin S., Staahl B.T., Alla R.K. and Doudna J.A. (2014) Enhanced homology-directed human genome engineering by controlled timing of CRISPR/Cas9 delivery. eLife 3, e04766 10.7554/eLife.04766 25497837 PMC4383097

[82] Fu Y., Foden J.A., Khayter C., Maeder M.L., Reyon D., Joung J.K. et al. (2013) High-frequency off-target mutagenesis induced by CRISPR–Cas nucleases in human cells. Nat. Biotechnol. 31, 822–826 10.1038/nbt.2623 23792628 PMC3773023

[83] Kleinstiver B.P., Pattanayak V., Prew M.S., Tsai S.Q., Nguyen N.T., Zheng Z. et al. (2016) High-fidelity CRISPR–Cas9 nucleases with no detectable genome-wide off-target effects. Nature 529, 490–495 10.1038/nature16526 26735016 PMC4851738

[84] Casini A., Olivieri M., Petris G., Montagna C., Reginato G., Maule G. et al. (2018) A highly specific SpCas9 variant is identified by *in vivo* screening in yeast. Nat. Biotechnol. 36, 265–271 10.1038/nbt.4066 29431739 PMC6066108

[85] Chen J.S., Dagdas Y.S., Kleinstiver B.P., Welch M.M., Sousa A.A., Harrington L.B. et al. (2017) Enhanced proofreading governs CRISPR–Cas9 targeting accuracy. Nature 550, 407–410 10.1038/nature24268 28931002 PMC5918688

[86] Lee J.K., Jeong E., Lee J., Jung M., Shin E., Kim Y. et al. (2018) Directed evolution of CRISPR–Cas9 to increase its specificity. Nat. Commun. 9, 1–10 10.1038/s41467-018-05477-x 30082838 PMC6078992

[87] Kim S., Bae T., Hwang J. and Kim J.-S. (2017) Rescue of high-specificity Cas9 variants using sgRNAs with matched 5′ nucleotides. Genome Biol. 18, 218 10.1186/s13059-017-1355-3 29141659 PMC5686910

[88] Singh D., Wang Y., Mallon J., Yang O., Fei J., Poddar A. et al. (2018) Mechanisms of improved specificity of engineered Cas9s revealed by single-molecule FRET analysis. Nat. Struct. Mol. Biol. 25, 347–354 10.1038/s41594-018-0051-7 29622787 PMC6195204

[89] Liu J.-J., Orlova N., Oakes B.L., Ma E., Spinner H.B., Baney K.L.M. et al. (2019) Casx enzymes comprise a distinct family of RNA-guided genome editors. Nature 566, 218–223 10.1038/s41586-019-0908-x 30718774 PMC6662743

[90] Cameron P., Coons M.M., Klompe S.E., Lied A.M., Smith S.C., Vidal B. et al. (2019) Harnessing type I CRISPR–Cas systems for genome engineering in human cells. Nat. Biotechnol. 37, 1471–1477 10.1038/s41587-019-0310-0 31740839

[91] Qi L.S., Larson M.H., Gilbert L.A., Doudna J.A., Weissman J.S., Arkin A.P. et al. (2013) Repurposing CRISPR as an RNA-guided platform for sequence-specific control of gene expression. Cell 152, 1173–1183 10.1016/j.cell.2013.02.022 23452860 PMC3664290

[92] Bikard D., Jiang W., Samai P., Hochschild A., Zhang F. and Marraffini L.A. (2013) Programmable repression and activation of bacterial gene expression using an engineered CRISPR–Cas system. Nucleic Acids Res. 41, 7429–7437 10.1093/nar/gkt520 23761437 PMC3753641

[93] Kampmann M. (2018) CRISPRi and CRISPRa screens in mammalian cells for precision biology and medicine. ACS Chem. Biol. 13, 406–416 10.1021/acschembio.7b00657 29035510 PMC5886776

[94] Dove S.L. and Hochschild A. (1998) Conversion of the ω subunit of *Escherichia coli* RNA polymerase into a transcriptional activator or an activation target. Genes Dev. 12, 745–754 10.1101/gad.12.5.745 9499408 PMC316573

[95] Komor A.C., Kim Y.B., Packer M.S., Zuris J.A. and Liu D.R. (2016) Programmable editing of a target base in genomic DNA without double-stranded DNA cleavage. Nature 533, 420 10.1038/nature17946 27096365 PMC4873371

[96] Nishida K., Arazoe T., Yachie N., Banno S., Kakimoto M., Tabata M. et al. (2016) Targeted nucleotide editing using hybrid prokaryotic and vertebrate adaptive immune systems. Science 353, aaf8729 10.1126/science.aaf8729 27492474

[97] Gaudelli N.M., Komor A.C., Rees H.A., Packer M.S., Badran A.H., Bryson D.I. et al. (2017) Programmable base editing of A•T to G•C in genomic DNA without DNA cleavage. Nature 551, 464–471 10.1038/nature24644 29160308 PMC5726555

[98] Li X., Wang Y., Liu Y., Yang B., Wang X., Wei J. et al. (2018) Base editing with a Cpf1–cytidine deaminase fusion. Nat. Biotechnol. 36, 324–327 10.1038/nbt.4102 29553573

[99] Komor A.C., Zhao K.T., Packer M.S., Gaudelli N.M., Waterbury A.L., Koblan L.W. et al. (2017) Improved base excision repair inhibition and bacteriophage Mu Gam protein yields C:G-to-T:A base editors with higher efficiency and product purity. Sci. Adv. 3, eaao4774 10.1126/sciadv.aao4774 28875174 PMC5576876

[100] Zuo E., Sun Y., Wei W., Yuan T., Ying W., Sun H. et al. (2019) Cytosine base editor generates substantial off-target single-nucleotide variants in mouse embryos. Science 364, 289–292 10.1126/science.aav9973 30819928 PMC7301308

[101] Jin S., Zong Y., Gao Q., Zhu Z., Wang Y., Qin P. et al. (2019) Cytosine, but not adenine, base editors induce genome-wide off-target mutations in rice. Science 364, 292–295 10.1126/science.aaw7166 30819931

[102] Grünewald J., Zhou R., Garcia S.P., Iyer S., Lareau C.A., Aryee M.J. et al. (2019) Transcriptome-wide off-target RNA editing induced by CRISPR-guided DNA base editors. Nature 569, 433–437 10.1038/s41586-019-1161-z 30995674 PMC6657343

[103] Rees H.A., Wilson C., Doman J.L. and Liu D.R. (2019) Analysis and minimization of cellular RNA editing by DNA adenine base editors. Sci. Adv. 5, eaax5717 10.1126/sciadv.aax5717 31086823 PMC6506237

[104] Anzalone A.V., Randolph P.B., Davis J.R., Sousa A.A., Koblan L.W., Levy J.M. et al. (2019) Search-and-replace genome editing without double-strand breaks or donor DNA. Nature 576, 149–157 10.1038/s41586-019-1711-4 31634902 PMC6907074

[105] Peters J.E., Makarova K.S., Shmakov S. and Koonin E.V. (2017) Recruitment of CRISPR–Cas systems by Tn7-like transposons. Proc. Natl Acad. Sci. U.S.A. 114, E7358–E7366 10.1073/pnas.1709035114 28811374 PMC5584455

[106] Faure G., Shmakov S.A., Yan W.X., Cheng D.R., Scott D.A., Peters J.E. et al. (2019) CRISPR–cas in mobile genetic elements: counter-defence and beyond. Nat. Rev. Microbiol. 17, 513–525 10.1038/s41579-019-0204-7 31165781 PMC11165670

[107] Strecker J., Ladha A., Gardner Z., Schmid-Burgk J.L., Makarova K.S., Koonin E.V. et al. (2019) RNA-guided DNA insertion with CRISPR-associated transposases. Science 365, 48–53 10.1126/science.aax9181 31171706 PMC6659118

[108] Klompe S.E., Vo P.L.H., Halpin-Healy T.S. and Sternberg S.H. (2019) Transposon-encoded CRISPR–Cas systems direct RNA-guided DNA integration. Nature 571, 219–225 10.1038/s41586-019-1323-z 31189177

[109] Schrödinger L.LC. (2019) The PyMOL Molecular Graphics System, Version 2.3. 2019

